# Adsorption Materials and Their Applications

**DOI:** 10.3390/ma18235370

**Published:** 2025-11-28

**Authors:** Bożena Czech

**Affiliations:** Department of Radiochemistry and Environmental Chemistry, Faculty of Chemistry, Maria Curie-Skłodowska University, Lublin, Pl. M. Curie-Skłodowskiej 3, 20-031 Lublin, Poland; bozena.czech@mail.umcs.pl

## 1. Adsorption Materials

The rise in the development of analytical methods has resulted in an increased number of substances being detected in environmental matrices, known as emerging pollutants [1]. Although they are detected at relatively low concentrations, their persistence and frequent bioactivity make them refractory pollutants [2]. Existing water and wastewater treatment methods are ineffective for their removal [3], necessitating the development of new effective and environmentally friendly methods. Among various proposed techniques for the treatment of air, soil, water, and wastewater, adsorption seems to be the most promising solution, as it is cheap, effective, and easy to use [4].

Growing global pressure on freshwater resources, combined with rapid industrialization and increasing chemical consumption, has accelerated research on new materials for the adsorption, degradation, detection, and removal of pollutants from air, soil, water, and wastewater [3,5,6]. The diversity of contaminants, including metals [7], radionuclides [8], pharmaceuticals [9], pesticides [10], dyes [11], etc., requires tailored remediation strategies, and designing effective and environmentally friendly materials is crucial in the modern era [12,13,14,15]. In addition for being effective and cheap, adsorption does not require harsh conditions [16]. Furthermore, the transformation of wastes into precious products such as sorbents meets the requirements of the circular economy and sustainable development, and enables the realization of several Sustainable Development Goals [17]. Engineered materials designed for the removal of toxic and refractory pollutants may help address several environmental issues. The necessity and importance of studies on this topic are reflected in the 40 articles published in the two editions of this Special Issue.

## 2. Current Studies

The current studies in this field present wide-ranging advances in adsorbent synthesis, mechanochemical activation, porous-material characterization, computational modeling, bio-derived carbons, polymeric sorbents, quantum dots, and mesoporous structures. Collectively, they illustrate the richness and complexity of contemporary research in adsorption science and nanomaterial engineering. Among the papers submitted to this Special Issue, the major findings and contributions can be grouped into the following themes: (i) nanomaterials and engineered sorbents, (ii) bio- and waste-derived carbons, (iii) inorganic and mineral sorbents, (iv) polymeric and hybrid adsorbents, (v) dye and pharmaceutical removal, (vi) heavy-metal and radionuclide adsorption, (vii) computational modeling and porous-structure analysis, and (viii) innovative fabrication routes such as microwave-assisted synthesis and mechanochemical activation. By examining the insights provided by these studies, we highlight emerging trends and avenues for future research in the field of adsorption-based water treatment.

## 3. Innovative Trends

Mechanochemical activation of biomass or waste precursors represents a solvent-free, energy-efficient method that enhances porosity and functional-group density, as demonstrated in sawdust-derived carbons. Microwave irradiation significantly accelerates carbonization, activation, and nanoparticle formation, producing high-performance adsorbents in minutes rather than hours. Crosslinked polymers, polymer composites, and impregnated polymeric beads indicate the adaptability of polymer chemistry to produce targeted adsorbents for metals, dyes, and pharmaceuticals. Across these diverse studies, several major trends emerge:

1. Sustainability and circular economy:

Many adsorbents originate from agricultural waste, industrial residues, or natural materials, reflecting the shift toward low-cost and eco-friendly sorbents.

2. Functionalization and surface chemistry control:

Tailoring surface groups (amine, sulfur, nitrogen, quaternary ammonium) is crucial for enhancing selectivity and adsorption strength.

3. Nanostructure engineering:

Mesoporosity, surface area, and hierarchical pore systems are key determinants of performance, as is evident across carbons, polymer networks, and mineral sorbents.

4. Hybrid multifunctional materials:

Materials such as quantum dots and magnetic nanospheres combine sensing, adsorption, and separation of pollutants from different matrices.

5. Advanced characterization and modeling:

DFT simulations, tomography, and machine learning help elucidate adsorption mechanisms and accelerate material development.

6. Performance in real or complex systems:

Several articles emphasize the competitive effects of multi-component adsorption and validate its efficacy in industrial solutions, indicating its potential for real-world applications.

## 4. Summary

The 40 studies submitted to the two editions of the Special Issue “Adsorption Materials and Their Applications” represent significant progress in the development and characterization of novel adsorbents for environmental applications. Advances in this field span biochar production, ordered mesoporous materials, polymeric sorbents, magnetic nanoparticles, and computationally guided designs. Collectively, this body of work strengthens our understanding of how composition, structure, and surface chemistry influence adsorption processes across pollutants ranging from pharmaceuticals and dyes to heavy metals and radioactive species. Future research should integrate real-time monitoring, pilot-scale testing, and life-cycle analysis while exploring hybrid materials that unify adsorption, catalysis, and sensing. The convergence of green chemistry, nanotechnology, and advanced modeling will continue to drive innovation in sustainable water purification and resource recovery.

## Data Availability

Not applicable.
